# Menopausal Symptoms, Postural Balance, and Functional Mobility in Middle-Aged Postmenopausal Women

**DOI:** 10.3390/diagnostics11122178

**Published:** 2021-11-24

**Authors:** João Espírito Santo, Agustín Aibar-Almazán, Antonio Martínez-Amat, Nuno Eduardo Marques de Loureiro, Vânia Brandão-Loureiro, María Leyre Lavilla-Lerma, Fidel Hita-Contreras

**Affiliations:** 1 Escola Superior de Saúde Atlântica, 2730-036 Barcarena, Portugal; espirito94@gmail.com; 2 Escola Superior de Educação, Instituto Politécnico de Beja, 7800-295 Beja, Portugal; nloureiro@ipbeja.pt (N.E.M.d.L.); vloureiro@ipbeja.pt (V.B.-L.); 3 Department of Health Sciences, Faculty of Health Sciences, University of Jaén, 23071 Jaén, Spain; amamat@ujaen.es (A.M.-A.); llavilla@ujaen.es (M.L.L.-L.); fhita@ujaen.es (F.H.-C.)

**Keywords:** postural control fall risk, menopausal symptoms, functional balance

## Abstract

The aim of the present study was to determine the associations between the severity of the menopausal symptoms and postural balance and functional mobility in middle-aged postmenopausal women. A cross-sectional study was performed (171 participants, 57.18 ± 4.68 years). Severity of the menopausal symptoms (on the Menopause Rating Scale), postural balance (stabilometric platform) with eyes open and closed, and functional mobility (timed up and go test) were determined. A multivariate linear regression was performed, with body mass index, waist to hip ratio, age and fall history as possible confounders. Our findings showed that a greater severity of the menopausal symptoms at a psychological level was associated, under both eyes open and closed conditions, with worse postural control assessed by the length of the stabilogram (adjusted R^2^ = 0.093 and 0.91, respectively), the anteroposterior center of pressure displacements (adjusted R^2^ = 0.051 and 0.031, respectively) and the center of pressure velocity (adjusted R^2^ = 0.065 for both conditions). Older age was related to greater mediolateral displacements of the center of pressure with eyes open and closed (adjusted R^2^ = 0.45 and 0.58, respectively). There were no associations between the menopausal symptoms’ severity and functional mobility. We can conclude that a greater severity of psychological menopausal symptoms was independently associated with worse postural balance in middle-aged postmenopausal women.

## 1. Introduction

Menopause is defined as the permanent cessation of menstruation, confirmed after twelve consecutive months of amenorrhea. Menopause is characterized by physiological, psychosocial, and sociological changes associated with the reduction in ovarian function [1]. Menopausal symptoms include vasomotor symptoms such as hot flashes and night sweats, physical and mental fatigue, sleep problems and urogenital symptoms such as vaginal dryness and bladder and sexual dysfunctions [2,3]. It has also been shown that depression and anxiety symptoms, which are very prevalent in middle-aged women, increase during the climacteric period [4,5]. The menopause transition is also associated with increased body weight and changes in body composition [6]. In fact, estrogen decline has been linked to increased visceral fat and central adiposity [7]. 

Falls are a major public health problem, being the second leading cause of unintentional injury deaths worldwide [8], thus, it is important to give priority to fall-related research to determine effective policies aimed at fall prevention [9]. Age is one of the most important fall risk factors. Older people have the highest risk of fall-related injury and death, and this risk increases with age [8]. Nevertheless, it has been reported that the number of co-morbidities increased the risk of a fall in women over 40 years old, and this risk escalated with additional co-morbidities if they were over 60 years [10]. This greater risk of falling, as well as the presence of menopause-related decreased bone mineral density and strength, increases the number of fractures and fall-related injuries, making falls a major health concern in postmenopausal women [11].

Altered balance and gait have been recognized as important fall risk factors [12]. It has been reported that the menopause transition is associated with balance deterioration [1], and that estrogen treatment increased balance performance in postmenopausal women [13]. Postural instability is associated with the risk of falling, and stabilometric parameters have been shown to predict falls in postmenopausal women aged 50–65 years old [14]. In the menopause, there is an increase in intra-abdominal fat and total body weight [15], and aged-related loss of muscle mass and strength accelerate [16], and all these changes may affect functional mobility. Functional mobility is an adequate method to assess dynamic balance when performing activities during daily life, and thus, it is a key factor for preventing not only falls but also disability and dependency [17,18]. The timed up and go (TUG) test [19] is one of a range of measures identified in clinical guidelines as a possible screening tool to evaluate gait and balance functions and to identify older people at risk of falling [20].

The goal of the present study was to determine the possible associations between the impact of the menopausal symptoms and postural balance and functional mobility in middle-aged postmenopausal women. We hypothesized that a greater impact of the menopausal symptoms is associated with worse stabilometric parameters and longer times in performing the TUG test. 

## 2. Materials and Methods

### 2.1. Study Participants

An analytical cross-sectional study was conducted on 171 postmenopausal women. Participants were recruited by contacting the staff of several associations of postmenopausal women from Granada and Jaén (Spain). This study was approved by the Research Ethics Committee of the University of Jaén, Spain (OCT.18/4.PRY). All participants gave their written informed consent to participate in this study, which was conducted in accordance with the Declaration of Helsinki, good clinical practices, and all applicable laws and regulations. Inclusion criteria were: 45–65 years old, at least 12 months of amenorrhea, being able to understand the instructions and complete the questionnaires, and willing to provide their written informed consent to participate in the study. Exclusion criteria were being under hormonal replacement therapy, taking vestibular sedatives or other central nervous system depressants, suffering from any chronic and/or severe medical diseases or any neuropsychiatric disorder that could limit balance and physical activity (i.e., vestibular or neurological conditions).

### 2.2. Outcomes

#### 2.2.1. Sociodemographic and Anthropometric Data 

All women were questioned by well-trained interviewers, who collected demographic and clinical data such as age, occupational status, education and marital status, and the number of falls experienced in the previous 12 months (history of falls). Women were classified as fallers if they experienced one or more falls in the last year. A fall was defined as unintentionally coming to rest on the ground, floor, or other lower levels [21]. Body mass index (BMI) was obtained by dividing body weight (kg) obtained with a 100 g–130 kg precision digital weight scale (Tefal), by height (m^2^) calculated with an adult height scale (Asimed). A 1.5 m flexible tape was used to evaluate waist and hip circumferences. Waist to hip ratio (WHR) was calculated by dividing waist circumference (cm) by hip circumference (cm). As for BMI, values <25 indicated normal weight, between 25 and <30 overweight, and ≥30 obesity [22]. Regarding WHR, values <0.76 indicated a gynoid pattern of body fat distribution, between 0.76 and 0.86 a uniform pattern, and ≥0.86 an android pattern [23].

#### 2.2.2. Postural Balance

In order to assess postural balance, we used a FreeMED resistive multisensor platform (Sensor Medica, Rome, Italy) and the Free-Step Standard 3.0 software (Sensor Medica, Rome, Italy). The Romberg test was performed under eyes-open (EO) and eyes-closed (EC) conditions. Participants stood barefoot, with their feet at a 30° angle, heels 2 cm apart, with their arms at their sides. Each session lasted 30 s, with a 1-min interval between sessions. The following parameters related to the center of pressure (CoP) under each condition were obtained: velocity of CoP displacements (V, mm/s), length of the stabilogram (L, mm), and the root mean square amplitude of CoP in the mediolateral (RMSX) and anteroposterior (RMSY) directions (mm). These stabilometric variables were obtained under both EO and EC conditions.

#### 2.2.3. Functional Mobility

In order to assess functional mobility, the TUG test was used [19]. This test has been proven a sensitive and specific instrument for identifying community-dwelling adults who are at risk of falls [24]. For this test, participants had to rise from a seated position on a chair, walk three meters, turn around, return, and sit down again. The time required to complete this test was recorded. Longer times correspond to worse functional mobility. 

#### 2.2.4. Severity of the Menopausal Symptoms

The Menopause Rating Scale (MRS) was used to assess the severity and the impact of menopausal symptoms on quality of life [25,26]. This scale has 11 items (scored from 0–4) organized in a total score and three domains: somatic (four items), psychological (four items), and urogenital (three items). Greater scores reflect a greater severity of menopausal symptoms. Values equal to or greater than 17 (total score), and 9, 7, and 4 for the somatic, psychological, and urogenital domains, respectively, indicate severity of the symptoms.

### 2.3. Sample Size Calculation

According to Concato et al. [27], in a multivariate lineal regression model at least 20 subjects per event are required for an adequate sample size. Four independent variables (MRS somatic, psychological, and urogenital domains, and the MRS total score), together with four possible confounders (age, BMI, WHR, and the history of falls) were used in this study, and hence, 160 participants were required for this analysis. The final number of participants was 171.

### 2.4. Data Analysis

Data management and analysis were performed with the SPSS statistical package for the social sciences for Windows (SPSS Inc., Chicago, IL, USA). Categorical variables were presented as frequencies and percentages, whereas continuous variables were described using means and standard deviations. The Kolmogorov–Smirnov test was used to evaluate normality. To evaluate the individual associations between stabilometric variables with MRS domains and total scores, as well as other confounders such as age, BMI, WHR and the history of falls, a bivariate correlation analysis was employed. A Student’s *t*-test was performed to analyze differences regarding the history of falls. In order to study the multivariate independent associations between variables, a multivariate linear regression model was used, with the postural control parameters as dependent variables. Those independent variables and confounders exhibiting significant results (*p* < 0.05) in the bivariate analysis and the Student’s *t*-test were included in the multivariate linear regression. In order to calculate the effect size coefficient of multiple determination in the linear models, we used adjusted-R^2^. According to Cohen [28], adjusted-R^2^ can be classified as insignificant when <0.02, small if between 0.02 and 0.15, medium if between 0.15 and 0.35, and large if >0.35. A 95% confidence level was used (*p* < 0.05).

## 3. Results

Sociodemographic and clinical characteristics of the participants are presented in Table 1. Of the 171 women (57.17 ± 4.71 years), 18.71% reported having experienced a fall in the last year. Mean values of BMI and WHR indicated overweight (only 16.2% were obese) and uniform body fat distribution pattern, respectively. All MRS scores were considered as non-severe.

In the analysis of the individual associations between postural control and the severity of menopause-related symptoms and hot flashes, (Table 2), higher values in all stabilometric variables except for RMSXEO, RMSYEO and RMSYEC were associated with a greater impact of the menopausal symptoms at the MRS total score (all *p* < 0.01), as well as with the psychological MRS domain or subscale (all *p* < 0.01), which was also related to RMSYEC (*p* < 0.05). A bigger impact of the symptoms at a somatic level was linked to elevated VEO (*p* < 0.01), LEO, VEC, LEC and RMSYEO (*p* < 0.05). No association between postural control and the MRS urogenital domain was found. As for the functional mobility assessed by the TUG test, the analysis did not show any statistical correlation with the severity of the menopausal symptoms.

As for the possible confounders (Table 3), older age was associated with increased mediolateral displacements of the COP with both eyes open and closed (*p* < 0.01). There were no differences regarding the history of falls for postural control or functional mobility.

The analysis of the independent associations performed by the linear regression (Table 4) showed that the impact of the menopausal symptoms at a psychological level was a significant predictor of poorer postural control. More precisely, it was independently associated with the length of the stabilogram under eyes open and closed (adjusted R^2^ = 0.093 and 0.91 respectively), the velocity of the CoP displacements with eyes open and closed (adjusted R^2^ = 0.065 for both conditions), as well as with the anteroposterior displacements of the CoP under eyes open (adjusted R^2^ = 0.051) and closed (adjusted R^2^ = 0.031). Finally, older age was independently related to worse postural control regarding mediolateral displacements of the CoP both with eyes open and closed (adjusted R^2^ of 0.45 and 0.58, respectively).

## 4. Discussion

The objective of the present study was to assess the associations between the severity of the menopausal symptoms and postural control and functional mobility in Spanish middle-aged postmenopausal women. Our findings suggest that, taking into account possible confounders such as age, BMI, WHR, and the history of falls, the impact of the menopausal symptoms at a psychological level was independently associated with worse postural balance. There were no associations between the severity of the menopausal symptoms and functional mobility.

Menopausal symptoms significantly affect the quality of life of middle-aged women [29]. It has been shown that, in women aged 40–64 years worldwide, 62%, 57%, and 50% reported muscle and joint pain, vasomotor symptoms, and sleep disorders, respectively [30]; items that are included in the MRS somatic domain. The results of the present study showed that the higher impact on quality of life was observed at a somatic level. In fact, the MRS somatic domain had the highest percentage of women with severe symptoms (MRS ≥ 9), followed by the urogenital, and finally the psychological subscale. This is in accordance with the findings described in a multicenter cross-sectional study conducted on postmenopausal women from 11 Latin American countries, although their percentages were lower than those found in the present study. This may be due because the mean age in the Núñez-Pizarro et al. study [31] was younger (52.5 ± 4.7 years) and 50.2% of the participants were in the early postmenopausal stage (≤5 years since menopause onset).

It has been reported that physical and mental fatigue may influence postural control in older adults [32]. Besides, mood states and anxiety have been linked to alterations in the sensory and motor systems of balance control in healthy subjects. [33] This association may be explained by the neural connections between the brain areas for emotional control and for controlling posture and balance [34]. When analyzing the independent associations between the severity of the menopausal symptoms and postural control, our results showed that only a greater impact of the psychological symptoms was related to worse postural controls. These findings are in accordance with those previously described, since fatigue, anxiety, and depression are included in the MRS psychological domain. More precisely, these associations were found with all the posturographic parameters studied except for mediolateral displacements of the CoP, which was only related to older age. This could be explained because, according to the inverted pendulum model, the mediolateral postural sway is associated with the hip strategy to maintain the standing position, which is mainly adopted by older adults [35]. Given that the participants of this study were under 65 years old (mean age of 57.18 years), it is possible that most of them adopt the ankle strategy, which affects the anteroposterior but not the mediolateral displacements of the CoP, and thus, the lower mean values of the latter may limit its association with the menopausal psychological symptoms. 

During the menopause transition, women are predisposed to experience musculoskeletal pain [36], which is associated with decreased physical activity and mobility. As for the psychological factors, depression, but not anxiety, has been previously related to poorer functional mobility and described in women aged 60 years and over [37]. On the other hand, Ercan et al. [38] described that self-perceived fatigue was linked with functional mobility in middle-aged obese women [39]. The results of the present study regarding functional mobility do not show any significant associations with the MRS total score and the domains. This could be due to the fact that the times in performing the TUG test were lower, and thus reflected better functional mobility than those of these previous studies.

Obesity is considered as a fall risk factor in people aged 60 years and older [40]. It has recently been demonstarted that obesity, regardless of the presence of metabolic dysfunction, increases the risk of poor physical performance as assessed with several tests, including TUG [41]. However, a study conducted on 5970 Spanish people aged ≥65 years (European Health Survey 2014) concluded that there was no association between BMI and the degree of functional limitation [42]. On the other hand, higher BMI and WHR have been related to worse postural balance [43], and several adiposity measures have been shown to be associated with reduced postural balance, where waist circumference obtained the strongest association [44]. The results of this study did not reveal any associations between postural control and functional mobility with either BMI nor WHR. This may be explained because the WHR and BMI mean scores indicated uniform body fat distribution and overweight (with only 16.2% being obese), respectively.

The history of falls has been described as an important fall risk factor. Older adults who experienced one or more falls are more likely to fall again, and it is considered an essential part in the screening for falls [20]. Moreover, the history of falls is associated with the fear of falling again, which is linked to other negative consequences apart from falling, such as restriction or avoidance of activities of daily living, diminished social contact and poor quality of life [45]. As for postural control, Merlo et al. [46] described that the history of falls was associated with worse posturographic parameters assessed under different visual, proprioceptive, and cognitive conditions; however, we could not confirm this association. On the other hand, the association between the history of falls and functional mobility is not conclusive in the literature. TUG test times and a history of falls have been found to be related, but the clinical relevance of this association is limited [47]. Nevertheless, Asai et al. [48] found that slower time in the TUG test were related to fall history among independent community-dwelling older adults, while Kim et al. [49] reported that the TUG test score did not show a statistical relation to the history of falls. The findings of the present study did not show an association between the history of falls and both functional mobility and postural balance. This may be due to the younger mean age of the participants in the present study, and to the fact that TUG test mean time was 7.86 ± 1.41 s, which is lower than that described as a cutoff point to discriminate people at fall risk [16]. 

## 5. Limitations

Some limitations to our study should be noted. The cross-sectional nature of the study design does not allow establishing causal relationships. Moreover, this study was carried out in postmenopausal women recruited from a specific geographical area, and thus, any generalization of the results should be limited to people with similar characteristics to those of our sample. Although the validity and reliability of the TUG test has been shown in different populations regardless of age [50], the participants of the present study showed a mean score that can be considered as low, which may be responsible for the lack of significant differences. The history of falls was obtained by interviews and self-reports that attempted to identify the number of falls retrospectively, thus causing recall bias that may have influenced the estimated frequency of falls. Future studies should consider exploring prospective designs for a more diverse population, using other tests other than the TUG to assess functional mobility (i.e., gait speed or multiple sit-to-stands), and employing a diary to record the number of falls in order to avoid recall bias.

## 6. Conclusions

The results of this cross-sectional study indicated that, considering possible confounders, a higher impact of menopause-related psychological symptoms was a significant predictor of poorer postural control. More specifically:

- A greater severity of menopausal symptoms at a psychological level was associated, under both eyes open and closed conditions, with (i) longer length of the stabilogram, (ii) greater velocity of the CoP displacements, and (iii) larger anteroposterior displacements of the CoP.
- Older age was linked to worse postural control regarding mediolateral displacements of the CoP.
- Functional mobility was not related to the impact of menopausal symptoms on the quality of life.

## Figures and Tables

**Table 1:** Clinical and sociodemographic data of the participants.

		Participants (*n* = 171)	
		Mean	SD
Age (years)		57.18	4.68
Years since menopause onset		7.16	4.70
BMI		27.95	4.82
WHR		0.85	0.07
		Frequency	percentage
	None	11	6.43
	Primary	68	39.77
Education	Secondary	63	36.84
	University	29	16.96
Marital Status	Single	6	3.51
	Married/cohabiting	141	82.46
	Separated/divorced/widowed	24	14.03
History of Falls	No	139	81.29
	Yes	32	18.71
		Mean	SD
TUG test		7.86	1.41
MRS	Somatic	5.04	3.36
	Psychological	3.98	3.45
	Urogenital	3.50	2.96
	Total score	12.51	7.70

BMI: body mass index; MRS: Menopause Rating Scale; TUG: Timed Up and Go; SD: standard deviation; WHR: waist to hip ratio.

**Table 2:** Pearson’s correlations between postural control and MRS scores.

		MRS Somatic		MRS Psychological		MRS Urogenital		MRS Total Score	
		r	*p*-Value	r	*p*-Value	r	*p*-Value	r	*p*-Value
Eyes open	Velocity	0.21	0.007	0.26	0.001	0.02	0.821	0.21	0.006
	Length of the stabilogram	0.18	0.018	0.31	<0.001	0.04	0.564	0.23	0.002
	Mediolateral displacements	0.04	0.621	−0.02	0.808	−0.09	0.228	−0.03	0.723
	Anteroposterior displacements	0.18	0.018	0.23	0.003	0.06	0.468	0.20	0.008
Eyes closed	Velocity	0.17	0.025	0.26	0.001	0.03	0.668	0.20	0.008
	Length of the stabilogram	0.16	0.033	0.30	<0.001	0.06	0.418	0.23	0.002
	Mediolateral displacements	−0.06	0.458	−0.03	0.673	−0.09	0.242	−0.07	0.335
	Anteroposterior displacements	0.12	0.118	0.18	0.022	0.05	0.514	0.15	0.050
TUG test		0.13	0.091	0.08	0.302	0.07	0.365	0.12	0.121

MRS: Menopause Rating Scale; r: Pearson’s correlation coefficient; TUG: Timed Up and Go.

**Table 3:** Pearson’s correlations between postural control and age, BMI and WHR.

		Age		BMI		WHR	
		r	*p*-Value	r	*p*-Value	r	*p*-Value
Eyes open	Velocity	0.14	0.067	−0.01	0.899	0.02	0.832
	Length of the stabilogram	0.09	0.229	0.00	0.970	0.01	0.885
	Mediolateral displacements	0.21	0.005	−0.05	0.526	−0.02	0.842
	Anteroposterior displacements	0.09	0.260	−0.04	0.612	0.06	0.421
Eyes closed	Velocity	0.12	0.131	−0.00	0.989	0.01	0.908
	Length of the stabilogram	0.08	0.299	0.01	0.921	−0.01	0.943
	Mediolateral displacements	0.24	0.002	0.03	0.732	0.02	0.801
	Anteroposterior displacements	0.11	0.149	−0.07	0.354	0.03	0.717
TUG test		0.01	0.883	0.05	0.562	0.03	0.741

BMI: Body Mass Index; SD: standard deviation; r: Pearson’s correlation coefficient; TUG: Timed Up and Go; WHR: waist to hip ratio.

**Table 4:** Multivariate linear regression analyses.

		B	Beta	t	95% IC		*p*-Value
VEO	MRS psychological	0.499	0.256	0.001	0.212	0.785	0.001
LEO	MRS psychological	28.215	0.305	0.000	14.854	41.577	0.000
RMSXEO	Age	0.009	0.213	0.005	0.003	0.015	0.005
RMSYEO	MRS psychological	0.010	0.226	0.003	0.003	0.016	0.003
VEC	MRS psychological	0.541	0.256	0.001	0.230	0.853	0.001
LEC	MRS psychological	30.934	0.301	0.000	16.077	45.791	0.000
RMSXEC	Age	0.009	0.240	0.002	0.004	0.015	0.002
RMSYEC	MRS psychological	0.008	0.175	0.022	0.001	0.015	0.022

EO: eyes open; EC: eyes closed; L: length of the stabilogram; MRS: Menopause Rating Scale; RMSX: root mean square of the mediolateral displacements of the center of pressure; RMSY: root mean square of the anteroposterior displacements of the center of pressure; S: sway area covered by the center of pressure; TUG: timed up and go test; V: velocity of the center of pressure displacements.

## Data Availability

The data shown in this study are available upon request from the corresponding author. The data is not available to the public, since taking into account the sensitive nature of all the questions asked in this study, all participants were guaranteed that the data obtained would be confidential and would not be shared.
